# Progressive Multifocal Leukoencephalopathy in Systemic Lupus Erythematosus: A Consequence of Patient-Intrinsic or -Extrinsic Factors?

**DOI:** 10.3390/jcm12216945

**Published:** 2023-11-06

**Authors:** Evgenia Emmanouilidou, Despoina Kosmara, Efrosini Papadaki, Vasileios Mastorodemos, Pantelis Constantoulakis, Argyro Repa, Georgia Christopoulou, Christina Kalpadakis, Nestor Avgoustidis, Konstantinos Thomas, Dimitrios Boumpas, Prodromos Sidiropoulos, George Bertsias

**Affiliations:** 1 Rheumatology and Clinical Immunology, University Hospital of Heraklion and University of Crete Medical School, 71500 Heraklion, Greece; med5p1060169@med.uoc.gr (E.E.); despkosm@gmail.com (D.K.);; 2 Institute of Molecular Biology and Biotechnology, Foundation for Research and Technology—Hellas, 71110 Heraklion, Greece; 3 Department of Radiology, University Hospital of Heraklion and University of Crete Medical School, 71500 Heraklion, Greece; 4 Computational Bio-Medicine Laboratory, Institute of Computer Science, Foundation for Research and Technology—Hellas, 71110 Heraklion, Greece; 5 Department of Neurology, University Hospital of Heraklion, 71110 Heraklion, Greece; 6 Genotypos Science Labs Medical SA, 11528 Athens, Greece; 7 Department of Laboratory Hematology, University Hospital of Heraklion and University of Crete Medical School, 71500 Heraklion, Greece; 8 4th Department of Internal Medicine, National and Kapodistrian University of Athens School of Medicine, Attikon University General Hospital, 12462 Chaidari, Greece; 9 Laboratory of Autoimmunity and Inflammation, Center of Clinical, Experimental Surgery and Translational Research, Biomedical Research Foundation Academy of Athens, 11527 Athens, Greece

**Keywords:** polyomavirus, lymphopenia, immunodeficiency, GATA2, CDH7

## Abstract

Progressive multifocal leukoencephalopathy (PML) is a severe demyelinating disease of the central nervous system (CNS) caused by reactivation of the polyomavirus JC (JCV) typically in immunocompromised individuals. The risk of PML among rheumatic diseases may be higher for systemic lupus erythematosus (SLE), without, however, a clear association with the type and intensity of background therapy. We present the development and outcome of PML in a 32-year-old female lupus patient under mild immunosuppressive treatment, yet with marked B-cell lymphopenia in the peripheral blood and bone marrow (<1% of total lymphocytes). Despite treatment with the immune checkpoint inhibitor pembrolizumab, the patient showed progressive neurological and brain imaging deterioration and eventually died 15 months after PML diagnosis. To unveil possible underlying genetic liabilities, whole exome sequencing was performed which identified deleterious variants in *GATA2* and *CDH7* genes, which both have been linked to defective T- and/or B-lymphocyte production. These findings reiterate the possible role of disease-/patient-intrinsic factors, rather than that of drug-induced immunosuppression, in driving immune dysregulation and susceptibility to PML in certain patients with SLE.

## 1. Introduction

Progressive multifocal leukoencephalopathy (PML) is an opportunistic infection of the central nervous system (CNS) caused by reactivation of the polyomavirus John Cunningham (JC) virus (JCV) [1,2]. It occurs almost exclusively in immunocompromised individuals due to a variety of medical conditions such as malignancy, HIV infection, organ transplantation, primary immunodeficiency disorders, and treatment with heavy immunosuppressive agents. PML manifests with subacute neurologic deficits including altered mental status, motor deficits, limb ataxia, gait ataxia, and visual symptoms such as hemianopsia and diplopia [3]. Brain magnetic resonance imaging (MRI) reveals focal or multifocal white matter lesions, generally without mass effect, that do not conform to vascular territories [4]. Diagnosis is established by detecting DNA copies of the virus in the cerebrospinal fluid or brain tissue by Polymerase Chain Reaction (PCR) [3]. Histopathologic features suggestive of PML include demyelination, atypical astrocytes, and enlarged oligodendroglial nuclei [5].

Among various inflammatory rheumatic diseases, patients with systemic lupus erythematosus (SLE) are at a particularly increased risk for PML. By screening a large number of medical records in two tertiary hospitals in the United States, Kapoor et al. [6] reported a PML prevalence of 13–27 per 100,000 patients with SLE. Estimated incidence rates are in the range of 1 to 4 per 100,000 patients per annum, which is about 10 times higher than those in patients with rheumatoid arthritis [1,7]. Although several events of PML in SLE patients developed during or close to the administration of potent immunosuppressive drugs such as mycophenolate or the B-cell depleting agent rituximab [7,8], about half of the published cases occurred in the setting of minimal or no immunosuppressive treatment [5,7,9]. This observation has raised the hypothesis that lupus might inherently predispose patients to JCV infection/reactivation. 

To this end, no robust risk factors for SLE-associated PML have been identified so far, although such an endeavor is arduous due to the rarity of the disorder [7]. Nonetheless, previous studies have implicated lymphopenia, especially of the CD4+ T-cell compartment, as a predisposing factor [6,10,11,12]. Still, the majority of SLE patients with lymphopenia will not develop PML during their lifetime; therefore, the etiopathogenesis of JCV activation/PML in this context remains elusive. Another possibility that has not yet been explored is that specific genetically based immune aberrations accounting for lupus autoimmunity are linked to impaired host defense against JCV, a concept resembling the association between primary immunodeficiencies with autoimmune manifestations.

Herein, we report on a young SLE patient who developed PML while on mild background therapy and present the diagnostic and therapeutic challenges pertaining to this complication. In an attempt to unravel possible patient- or disease-related liabilities linked to PML, we performed peripheral blood immunophenotyping and analyzed the genetic make-up of the patients by means of whole exome sequencing. Our results hint towards the presence of multifactorial mechanisms underlying JCV activation and pathology. 

## 2. Materials and Methods

The study was conducted in accordance with the Declaration of Helsinki, with adherence to the STROBE (Strengthening the Reporting of Observational Studies in Epidemiology) methodological guidelines, and was approved by the University Hospital of Heraklion ethics committee (protocol numbers 38/14-11-2018 and 08/24-03-2021). Written informed consent for the patient data (clinical, imaging, genetic) to be published was given by the patient’s next of kin.

### 2.1. Blood and Bone Marrow Immunophenotyping 

Peripheral blood and bone marrow samples were obtained while the patient was receiving a stable prednisolone dose of 10 mg/day (withheld for 12 h before sample drawing) and prior to initiation of treatment with pembrolizumab. Aspirates were incubated for 20 min at 4 °C with monoclonal antibodies targeting standard lymphocyte membrane markers including CD3, CD4, CD8, CD19, CD16, CD56, CD57, and appropriate isotype controls (all from Beckman Coulter, CA, USA) in accordance with the standard immunophenotyping protocol of the University Hospital. The T-cell receptor (TCR)-Vβ repertoires of peripheral blood CD8+ T cells were also analyzed by flow cytometry using the IO Test Beta Mark TCR Vβ Repertoire Kit (Beckman Coulter, CA, USA). The kit provides mixtures of conjugated TCR Vβ antibodies, corresponding to 24 different specificities (about 70% coverage of normal human TCR Vβ repertoire). Cells were stained for 20 min at room temperature in the dark; then, they were lysed, washed, fixed with 0.5% formaldehyde, and acquired for flow cytometric analysis with a Beckman-Coulter Navios EX.

### 2.2. Genomic DNA Extraction and Exome Sequencing

DNA was extracted from peripheral blood following standard procedures (QIAamp DNA Blood Mini Kit, QIAGEN). Whole exome sequencing (WES) was performed as follows. In brief, exons, flanking intronic regions (±10 nucleotides), selected regulatory elements, and deep intronic regions of 21,285 genes targeting >98% of RefSeq and Gencode v28 regions of the expressed human genome were replicated from fragmented genomic DNA using the Twist Human Core Exome EF Multiplex Complete kit (Twist Bioscience, South San Francisco, CA, USA). The library was subsequently constructed by target capture selection following probe hybridization and then sequenced on a NextSeq500 platform (Illumina) to achieve a least a ×20 reading depth for approximately 99.3% of the targeted bases and uniform enrichment of target regions with a low duplicate read rate <10%.

### 2.3. Bioinformatics, Variant Calling, and Annotation

Bioinformatics analyses were performed by validated pipelines empowered by the SOPHiA DDM™ platform (Sophia Genetics, Lausanne, Switzerland). Sequences were aligned to the human genome reference GRCh37/hg19. Variant annotation is in accordance with the Human Genome Variation Society nomenclature. A gene panel was created including genes related to immunodeficiency according to the Human Phenotype Ontology (HPO) project (HP:0002721, last accessed on 12 October 2022), including the following: *ACD*, *ACP5*, *ACTB*, *ADA*, *ADA2*, *AGL*, *AICDA*, *AK2*, *AKT1*, *ALG1*, *AMN*, *ANTXR2*, *AP3D1*, *ARVCF*, *ARVCF*, *ATM*, *ATRX*, *BCL10*, *BCL11B*, *BCR*, *BCR*, *BLNK*, *BTK*, *BUB1B*, *CARD11*, *CARD9*, *CCDC47*, *CD19*, *CD247*, *CD28*, *CD3D*, *CD3E*, *CD3G*, *CD40*, *CD40LG*, *CD79A*, *CD79B*, *CD81*, *CDC42*, *CDCA7*, *CDH23*, *CEACAM3*, *CEACAM6*, *CFTR*, *CHD7*, *CLCA4*, *COMT*, *CORO1A*, *CPLX1*, *CR2*, *CREBBP*, *CRKL*, *CTBP1*, *CTC1*, *CTLA4*, *CTPS1*, *CUBN*, *CUL4B*, *CYBA*, *CYBB*, *DCLRE1C*, *DCTN4*, *DKC1*, *DNAJC21*, *DNMT3B*, *DOCK2*, *EDNRA*, *EFL1*, *EP300*, *EPG5*, *EXTL3*, *FCGR3A*, *FGFRL1*, *FOXN1*, *FRAS1*, *GATA2*, *GCLC*, *GP1BB*, *GSTM3*, *HBB*, *HELLS*, *HIRA*, *HMOX1*, *HYOU1*, *ICOS*, *IFNGR1*, *IFNGR2 IGLL1*, *IKBKB*, *IKBKG*, *IKZF1*, *IL12B*, *IL12RB1*, *IL21R*, *IL2RA*, *IL2RB*, *IL2RG*, *IL7R*, *IRAK4*, *IRF2BP2*, *IRF7*, *IRF8*, *ISG15*, *IVNS1ABP*, *JAK3*, *JMJD1C*, *KCNN4*, *KLLN*, *KNSTRN*, *LAMTOR2*, *LAT*, *LCK*, *LCP2*, *LETM1*, *LMNB2*, *LRBA*, *LRRC8A*, *LYST*, *MAGT1*, *MALT1*, *MAN2B1*, *MAPK1*, *MBTPS2*, *MCM10*, *MEIS2*, *MGAT2*, *MS4A1*, *MTHFD1*, *MYC*, *MYD88*, *NCF1*, *NCF2*, *NFE2L2*, *NFKB1*, *NFKB2*, *NHEJ1*, *NHP2*, *NOP10*, *NPM1*, *NSD2*, *ORAI1*, *PGM3*, *PIK3CA*, *PIK3CD*, *PIK3R1*, *PNP*, *POLE*, *PRKDC*, *PRPS1*, *PTEN*, *PTPRC*, *RAB27A*, *RAC2*, *RAG1*, *RAG2*, *RBCK1*, *RNF168*, *RREB1*, *RTEL1*, *SBDS*, *SDHB*, *SDHC*, *SDHD*, *SEC23B*, *SEC24C*, *SERPINA1*, *SH2D1A*, *SHANK3*, *SIK3*, *SKIV2L*, *SLC11A1*, *SLC26A9*, *SLC46A1*, *SLC6A14*, *SLC9A3*, *SMARCAL1*, *SP110*, *SPATA5*, *SRP54*, *STAT1*, *STK4*, *STX1A*, *TBCE*, *TBK1*, *TBX1*, *TCF3*, *TERT*, *TFRC*, *TGFB1*, *TICAM1*, *TINF2*, *TLR3*, *TNFRSF13B*, *TNFRSF13C*, *TNFRSF1B*, *TNFRSF4*, *TNFSF12*, *TRAF3*, *TTC37*, *TTC7A*, *TYK2*, *UFD1*, *UNC119*, *UNC93B1*, *UNG*, *USB1*, *USF3*, *USP8*, *WAS*, *WIPF1*, *WRAP53*, *XIAP*, *XRCC4*, *ZBTB24*, *ZNF699*.

For variant classification, data were evaluated from sources including but not limited to ExAC (r0.3.1), G1000 (v5.20130502), dbSNP (v155), GnomAD (r2.1), ClinVar (v20220416, www.ncbi.nlm.nih.gov/clinvar/, accessed on 12 October 2022), LOVD (www.lovd.nl/, accessed on 12 October 2022), Varsome Data Aggregator (https://varsome.com/, accessed on 12 October 2022), peer-reviewed literature, and in silico analyses; this was in accordance with the American College of Medical Genetics recommendations/guidelines (ACMG-2015 and ACGS-2020 guidelines).

### 2.4. Magnetic Resonance Imaging (MRI)

All brain MRI studies were performed on a clinical 1.5T whole-body superconducting imaging system (Vision/Sonata, Siemens/Erlangen), equipped with high-performance gradients (gradient strength: 40 mT/m, slew rate: 200 mT/m/ms) and a two-element circularly polarized head array coil. The conventional MR imaging protocol comprised the following sequences: (a) 3D T1-w (MPRAGE, time repetition (TR) 1570/time echo (TE) 1.73 ms, 1 mm^3^/1 NEX/160 axial slices), before and after intravenous Gadolinium administration, and axial sections of (b) TSE-T2-w turbo spin echo (TR/TE = 5000/98 ms), (c) TSE-FLAIR (TR/TE/TI = 9000/120/2600 ms), (d) diffusion weighted imaging (DWI) (TR/TE = 3400/100 ms, b-values = 0, 750, 1000), and (e) T2* FLASH 2D GRE (TR/TE = 625/14). Axial sections were acquired parallel to the plane connecting the anterior and posterior commissures (AC-PC lines), with a 4mm slice thickness. For all conventional scans, uniform geometry parameters were used (256 mm field of view and an acquisition matrix of 256 × 256). The MR angiography (3D-TOF MRA) was based on a clinically used standard 3D gradient echo sequence (flip angle of α = 25°, TE = 7 ms, TR = 40 ms, 1 slab with 144 slices per slab, and a voxel size of 0.4 mm^3^). For the non-invasive measurement of hemodynamic parameters, such as the cerebral blood volume (CBV) and cerebral blood flow (CBF), the T2* dynamic susceptibility contrast-perfusion MRI was performed utilizing a 2D single-shot multi-slice Gradient Echo–Echo Planar Imaging (GREEPI) sequence (TR/TE/flip angle (FA): 1500 ms/40 ms/30°, bandwidth (BW): 2442 Hz/pixel, echo spacing: 0.47 ms, and EPI factor 64). Finally, a single-voxel MR spectroscopy (TR/TE2000/135ms) was acquired for the estimation of the concentration of brain metabolites, such as Choline, Creatine, N-acetyl-aspartate (NAA), and lactate.

## 3. Results

### 3.1. Case Presentation

**Figure 1:**
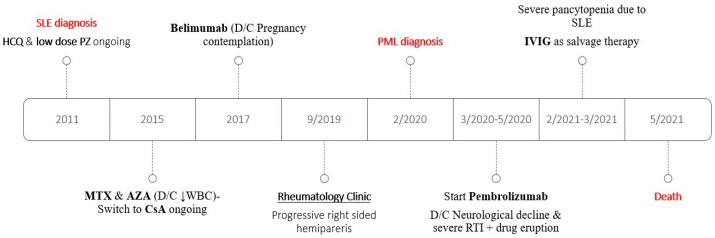
Sequence of events in the SLE patient. D/C: Discontinuation; HCQ: hydroxychloroquine; PZ: prednisolone; WBC: white blood cells; AZA: azathioprine; MTX: methotrexate; CsA: cyclosporine; RTI: respiratory tract infection; IVIG: intravenous immune globulin.

A young woman was diagnosed with SLE at the age of 24 years due to polyarthritis, malar rash, photosensitivity, leukopenia, lymphopenia, thrombocytopenia, non-scarring alopecia, Raynaud’s phenomenon, erythema nodosum, cutaneous vasculitis, and compatible serological abnormalities including antinuclear antibodies (ANA) at a titer of 1:640; anti-dsDNA, anti-Smith, and anti-SSB autoantibodies; low levels of serum complement fractions C3/C4; and positive direct Coombs. The rest of her medical history included hypothyroidism. During follow-up, she experienced non-organ/life-threatening disease activity with flares of arthritis, acute cutaneous lupus, skin vasculitis, and cytopenias with persistent lymphopenia in the range of 700–1100/μL. She was on stable hydroxychloroquine 400 mg/day, low-dose prednisolone (5 mg/day), and cyclosporine 300 mg/day. In the past, the patient had received methotrexate and azathioprine, both discontinued due to the worsening of leukopenia. Belimumab was also introduced for 6 months and then was stopped due to pregnancy contemplation (Figure 1).

In September 2019, she presented with a 3-month history of progressive right-sided weakness. On examination, right hemiparesis and right hemisensory impairment involving pinprick, temperature, and position sense were noted. She had no signs of active infection and the SLE Disease Activity Index (SLEDAI-2K [13]) was 6 due to arthritis and malar rash without serological activity. Her total lymphocyte count was 1000/μL.

**Figure 2:**
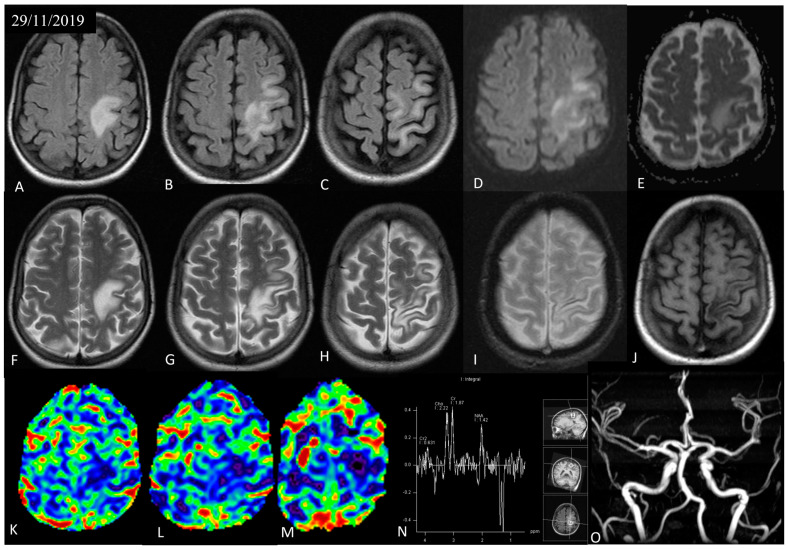
Initial brain MRI of the patient with axial fluid-attenuated inversion recovery (FLAIR) (**A**–**C**), diffusion weighted imaging (DWI) (**D**), apparent diffusion coefficient (ADC) map (**E**), T2 (**F**–**H**), gradient echo (GRE) (**I**), and post-Gadolinium T1 (**J**) sections. A large, hyperintense, non- enhancing white matter lesion was revealed in the left frontoparietal area, with patchy, moderate restricted diffusion, along with local thinning and hemosiderin deposition of the adjacent cortex. Cerebral blood flow (CBF) map (**K**–**M**) derived from dynamic susceptibility contrast (DSC)-perfusion MRI showed decreased cerebral blood flow (CBF) in the involved parenchyma. Single-voxel spectroscopy (TE = 135 ms) (**N**) placed at the region of interest revealed the reduction in the N-acetyl aspartate (NAA)-to-creatine ratio (0.76), without a significant increase in the choline-to-creatine ratio (1.2) and presence of an inverted lactate peak. Magnetic resonance angiography (MRA) (**O**) of the brain was normal.

Initial brain magnetic resonance imaging (MRI) revealed a large, non-enhancing, T2 hyperintense white matter lesion in the left frontoparietal area with patchy, moderate restricted diffusion, along with local thinning and hemosiderin deposition at the adjacent cortex (Figure 2). Perfusion MRI showed decreased cerebral blood flow of the involved parenchyma. Single-voxel spectroscopy placed at the region of interest revealed a reduction in the N-acetyl aspartate (NAA)-to-creatine ratio (0.76), indicative of neuronal loss, without a significant increase in the choline-to-creatine ratio (1.2) and presence of inverted lactate peak, suggestive of anaerobic glycolysis. At this point, diagnosis was oriented to the ischemic substrate of the lesion, possibly the result of lupus-related vasculitis or thromboembolic disease, although the differential diagnoses also included infection, demyelinating disease with atypical imaging findings, and low-grade malignancy. Brain magnetic angiography (MRA) was normal, thus excluding large-vessel vascular disease (Figure 2). 

Lumbar puncture yielded zero white blood cells, two red blood cells, normal glucose, and a mildly elevated protein level of 81 mg/dL (normal 15–45 mg/dL). IgG index was normal and there were type IV oligoclonal bands (i.e., identical in serum and in cerebrospinal fluid). The rest of the workup for infectious pathogens, thromboembolic disease, and antiphospholipid antibodies was negative.

**Figure 3:**
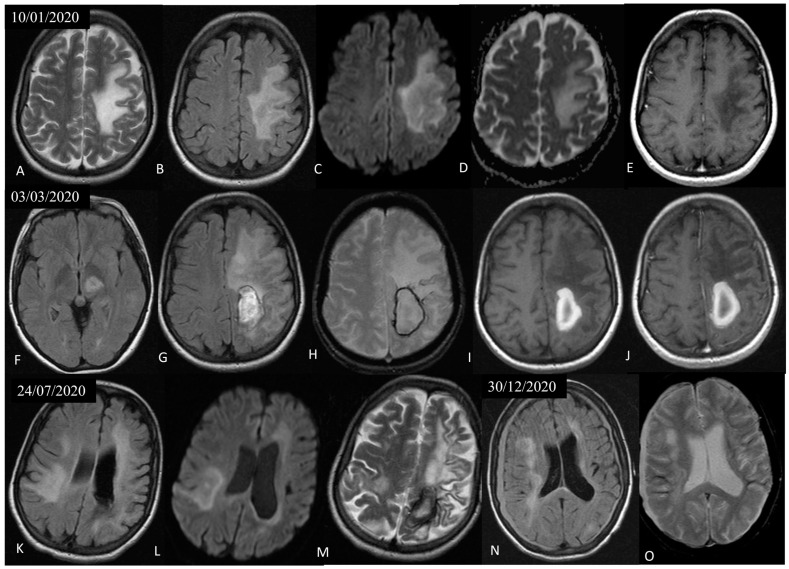
Axial T2 (**A**), FLAIR (**B**), DWI (**C**), ADC map (**D**), and post-Gadolinium T1 (**E**) sections of a follow-up brain MRI that was performed 1,5 months after the initial MRI, showed expansion of the pre-existing non-enhancing, T2 hyperintense, white matter lesion in the left frontoparietal area, with characteristic involvement of the U-fibers, lack of mass effect, and peripheral restricted diffusion, indicative of PML. Two months later, axial FLAIR (**F**,**G**), GRE (**H**), and pre- (**I**) and post-Gadolinium T1 (**J**) sections of a follow-up MRI showed further progression of the non-enhancing left frontoparietal lesion, a new lesion in the left thalamus, extending to the mesencephalon, and a subacute left parietal hematoma. Two more follow-up MRIs 5 months (axial FLAIR (**K**,**L**) and T2 (**M**)) and 10 months (axial FLAIR (**N**) and GRE (**O**)) later revealed new lesions in the right frontotemporal and parietal areas, with imaging characteristics also suggestive of PML, further loss of brain parenchyma and cortical hemorrhagic deposition in the left hemisphere, as well as bilateral frontoparietal subdural hematomas.

Soon after, the patient deteriorated with new-onset dysarthria and mixed aphasia. A new brain MRI showed expansion of the non-enhancing, white matter, T2 hyperintense lesions in the left frontoparietal area, with characteristic involvement of the U-fibers, lack of mass effect, and peripheral restricted diffusion, findings that raised high suspicion for PML (Figure 3). At this point, a second lumbar puncture was performed, in which PCR for JCV in the CSF was positive. Nonetheless, the lack of heavy treatment-related immunosuppression prompted us to perform a brain biopsy in order to exclude other pathologies such as lymphoma. Histological examination demonstrated extensive demyelination with macrophage-rich lesions (CD163+ foam-like cells) and oligodendroglia with enlarged, glassy nuclei, suggestive of polyoma virus infection [14]. 

During the following weeks, the patient showed progressive neurological decline. In an effort to revamp the immune system against JCV and based on published experience [15], she was started on intravenous pembrolizumab 200 mg every month. However, after three cycles of therapy, the patient developed a generalized drug eruption and was hospitalized in the intensive care unit due to a severe respiratory tract infection. Moreover, there was no clinical improvement and, therefore, treatment with pembrolizumab was ceased.

The patient continued to deteriorate with the appearance of global aphasia and left-sided hemiparesis. New brain MRIs showed progression of the pre-existing left frontoparietal lesion; new lesions in the left thalamus, extending to the mesencephalon and also to the right frontotemporal and parietal areas, with imaging characteristics suggestive of PML; further loss of brain parenchyma and cortical hemorrhagic deposition in the left hemisphere; as well as an intraparenchymal hematoma in the left parietal lobe and bilateral subdural hematomas (Figure 3). In the ensuring months, she was hospitalized multiple times due to recurrent episodes of aspiration pneumonia. Along with the neurologic decline, she gradually developed severe pancytopenia attributable to SLE, and as a rescue therapy, she received intravenous immune globulin (IVIG). However, her clinical status progressed and she eventually died 15 months after the diagnosis of PML.

### 3.2. Immunophenotyping Assessment

From the diagnosis of PML, the patient had an average total lymphocyte count of 700/μL with a nadir level of 200/μL. This prompted us to perform immunophenotyping by flow cytometry in the peripheral blood and bone marrow in order to estimate the abundances of individual immune cell subsets. The results shown in Table 1 suggested a marked reduction in CD19+ B cells in both sites (0.2–0.8% of lymphocytes) despite the fact that our patient had never received a B-cell depleting treatment. The CD4+ T-cell count in the peripheral blood was 175/μL (35% of lymphocytes), which is below the cytopenia threshold (300 cells/μL) set by the Centers for Disease Control and Prevention, although the relative proportion exceeded 20% of total T cells. Another notable finding was the expansion in CD3+CD8+CD57+ cells (26.9%) which corresponded to antigen-specific, immune-senescent, functionally competent memory/effector CD8+ T lymphocytes [16]. Indeed, the TCR-Vb clonality assessment by flow cytometry revealed the oligoclonal expansion (Vb16, Vb20) of blood CD8+ T cells.

### 3.3. Whole Exome Sequencing Analysis 

**Figure 4:**
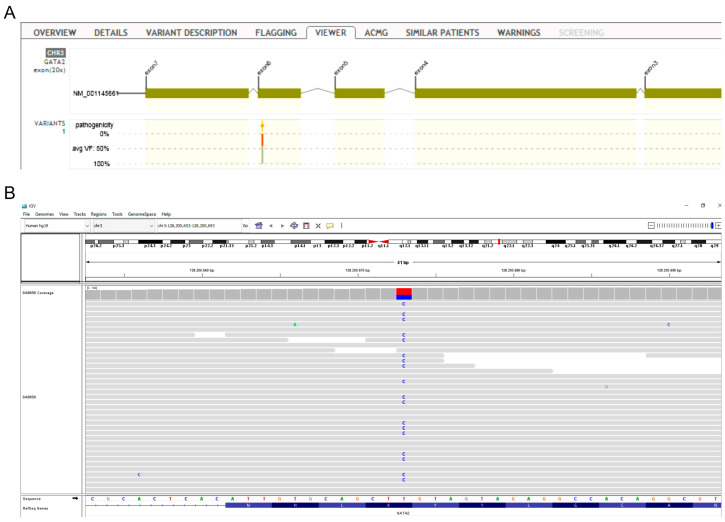
(**A**) *GATA2* structure. The red arrow indicates the c.1132A>G variant position in exon 6. (**B**) Integrative Genomics Viewer (IGV) screenshot showing the *GATA2* c.1132A>G variant.

WES revealed heterozygous mutations in two genes included in the immunodeficiency panel. Specifically, our patient carried the c.1132A>G substitution in the GATA Binding Protein 2 (GATA2) gene (Figure 4), of which there are no previous reports in the database of ClinVar and LOVD (Leiden Open Variation Database). According to the American College of Medical Genetics and Genomics (ACMG) criteria (PM1mod, PM2mod, and PP3sup), c.1132A>G is classified as a variant of uncertain clinical significance with strong indications of pathogenic influence (hot-VUS). The variant is located in a genetic region with an increased frequency of pathogenic mutations (hot spot), it is not detected in the population database (GnomAD), and in silico models predict that it may be harmful (aggregated score 0.99). Mutations in GATA2 have been linked to severe B-cell lymphopenia, cytopenias, bone marrow failure, and immunodeficiency [17], in particular, the autosomal dominant immunodeficiency 21 (MIM# 614172) [18].

**Figure 5:**
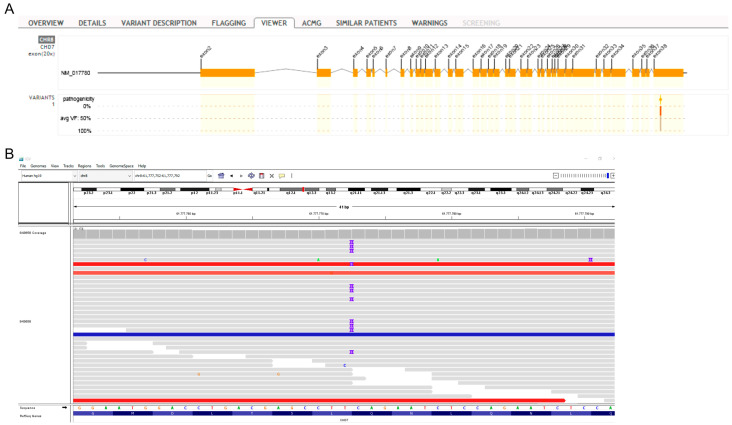
(**A**) *CHD7* structure. The red arrow indicates the c.8287_8295dup position in exon 38. (**B**) Integrative Genomics Viewer (IGV) screenshot showing the *CHD7* c.8287_8295dup variant.

Second, a duplication of nucleotides c.8287 to c.8295 (c.8287_8295dup) in exon 38 of the CHD7 gene (Figure 5) was identified. CHD7 encodes for the chromodomain helicase DNA-binding protein 7. The above mutation is reported as a variant of uncertain clinical significance in the database of ClinVar; however, according to the ACMG criteria (PM2mod, PP4mod), it is classified as a variant of unknown clinical significance with strong indications of pathogenic influence (hot-VUS). It is a rare mutation, which leads to the addition of amino acids without modifying the reading frame in a region that does not have repeats. CHD7 gene defects are associated with the CHARGE syndrome and hypogonadotropic hypogonadism 5 with or without hyposmia, which are inherited with an autosomal dominant manner [19,20]. In addition, CHD7 mutations may cause impairment of thymic development and thus low or absent T-cell production and, rarely, B-cell depletion [21]. 

## 4. Discussion

PML represents an uncommon yet dreadful complication in immunocompromised individuals including patients with autoimmune rheumatic diseases, leading to severe neurological deficit and/or fatal outcome [1,3,5]. Prompt and accurate identification of PML can be challenging, yet it is critical for proper patient management and minimization of compounding factors such as immunosuppressive therapies [22,23]. This is further emphasized in our case of a young SLE woman where the initial differential diagnoses guided by the first brain MRI were broad and included disease-related conditions (i.e., neuropsychiatric lupus with stroke-like presentation or cerebral vasculitis), infections, and malignant disorders such as lymphoma. From a neuroimaging standpoint, the findings that may facilitate the early recognition of PML include juxtacortical U-fiber involvement, DWI hyperintense rim, and punctate white matter T2 lesions (with or without contrast enhancement) [24,25]. Moreover, the specific patterns of brain involvement in PML and their differentiation from other pathologies are discussed elsewhere [14,23]. Clinical clues to alert physicians include newly appearing cognitive dysfunction or seizure disorder in SLE [23], especially in the context of heavy immunosuppressive treatment or failure to improve with glucocorticoids.

Contrary to multiple sclerosis where the majority of PML cases have been linked to the administration of natalizumab (anti-α4-integrin monoclonal antibody) [26], the association with the type or intensity of therapy has been less consistent in SLE. This observation, coupled with the excessive PML risk in lupus [1,7], has put forward the hypothesis that disease-intrinsic immune deregulation might contribute to JCV activation. In this regard, lymphopenia, especially of CD4+ T cells, has been proposed as a risk factor for PML in SLE [6,10,11,12] and other individuals [27]. Lymphopenia is considered a typical disease manifestation of lupus, although it may also occur as a side effect of administered immunosuppressive therapy. Notably, lymphopenia is often overlooked in clinical practice and there are no current established guidelines for the treatment of SLE patients presenting severe or persistent lymphopenia.

Although our patient exhibited a modest reduction in the absolute number of circulating T cells, we were stricken by the profound CD19+ B-cell lymphopenia in the absence of previous B-cell depleting treatment. B cells might have a dual role in PML pathogenesis: first, they impact the function and robustness of T-cell responses through cytokine production and cell-to-cell contact [28]; second, they can act as a viral reservoir and as a vector for JCV dissemination in the CNS. Thus, JCV can infect CD34+ hematopoietic precursor cells, glial cells, and B cells but not primary T cells [29]. The virus exists in two forms: a non-pathogenic, also called archetypal, virus and a neurotropic that contains a rearranged non-coding control region (NCCR) [30]. B lymphocytes express certain transcription factors which bind to the NCCR of the neurotropic form, therefore facilitating the generation, persistence, and dissemination of JCV [31]. 

The aforementioned lines of evidence provide some possible explanation for the enhanced susceptibility to PML following treatment with B-cell depleting agents such as rituximab [7,8,32]. Thus, it has been suggested that JCV spreading may occur during the B-cell repopulation phase following their prior drug-induced elimination [33]. In addition, by removing B cells, T-cell responses against JCV may be impaired [2]. 

Circumstantial evidence implicates genetic susceptibility to JCV activation and disease [34]. Notably, a recent study has identified specific genotype × drug exposure interactions that modify the risk for PML in patients with multiple sclerosis [35]. In our patient, WES analysis focusing on a gene panel linked to immunodeficiencies identified two variants (c.1132A>G substitution in *GATA2*, c.8287_8295dup in *CHD7*) likely ascribed to have a negative impact on the immune system. Interestingly, mutations in GATA2 have been associated with B-cell lymphopenia [17], which was a prominent characteristic in our patient. However, in the absence of mechanistic experimental studies and pending confirmation in additional lupus cohorts, no causal inferences can be drawn. Nonetheless, to our knowledge, no previous studies have probed the genetic background of SLE patients affected with PML, an endeavor that may be worth further investigation towards possible personalized risk assessment.

Based on previous published data and the expansion of immune-senescent, antigen-experienced CD8+ T cells in the peripheral blood of our patient, we attempted to awaken the anti-JCV T-cell response by administering the anti-PD1 checkpoint inhibitor pembrolizumab. Following the sentinel study by Cortese et al. [15], which was performed in an unselected patient population, a single case of SLE PML successfully managed with pembrolizumab was reported [36]. Unfortunately, our patient continued to progress and also developed signs suggestive of lupus flare; thus, pembrolizumab was discontinued. To this end, effective treatment of PML in non-HIV individuals such as those with SLE represents a major unmet need. Nonetheless, there is rationale to explore immune-directed modalities, and accordingly, the possible utility of exogenous IL-2 and IL-7 to expand the pool of CD4+ T cells has been proposed [12]. 

**Figure 6:**
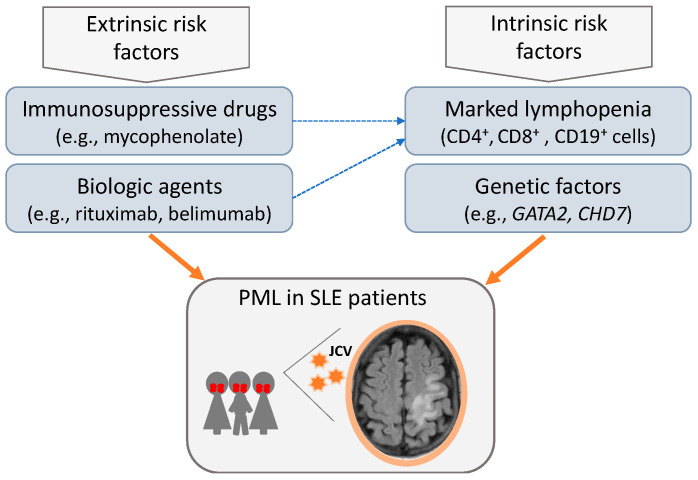
Disease-extrinsic and -intrinsic risk factors presumably linked to increased susceptibility for JC virus re-activation and PML in patients with SLE.

In conclusion, our study reiterates the multifactorial basis for increased susceptibility to JCV activation in certain patients with SLE, likely due to a complex interplay between genetic factors, immune deregulations, and drug-induced immunosuppression (summarized in Figure 6). Pending additional studies to disentangle the host–JC virus interactions in lupus, clinicians should be aware of this severe complication and have a high index of suspicion in patients presenting with persistent lymphopenia even under no heavy immunosuppression therapy.

## Figures and Tables

**Table 1:** Immunophenotyping results in peripheral blood and bone marrow ^1^.

Cellular Marker	Peripheral Blood (% Lymphocytes)	Bone Marrow (% Lymphocytes)
CD3+	99.5	98.7
CD3+ CD4+	35.0	51.0
CD3+ CD8+	57.0	40.0
CD4+ CD8+	3.6	5.2
CD19+	0.2	0.8
CD16+ CD56+ CD3−	0.1	0.1
CD16+ CD56+ CD3+	0.0	4.1
CD57+ CD8+ CD3+	26.9	15.4
CD57+ CD4+ CD3+	8.3	6.9

^1^ At the time of analysis, absolute total lymphocyte count in the peripheral blood was 500/μL.

## Data Availability

Data are available upon reasonable request.
